# The impact of insulin pump therapy to oxidative stress in patients with diabetic nephropathy

**DOI:** 10.1186/s40001-018-0304-2

**Published:** 2018-02-12

**Authors:** Xing-Guang Zhang, Yan-Qi Zhang, Qian-Peng Cheng, Yi Cao, Jian-Min Sun, Xiao-Feng Lv

**Affiliations:** 1 Department of Endocrinology, PLA Army General Hospital, Beijing, China; 2 China National Institute of Standardization, Beijing, China

**Keywords:** Diabetic nephropathy, Oxidative stress, Insulin pump intensive therapy

## Abstract

**Background:**

The oxidative stress resulting from increased production of ROS plays a crucial role in the development of diabetic complications. We aim to explore the relationships between oxidative stress, diabetic nephropathy (DN) and short-term insulin pump intensive therapy (insulin therapy).

**Methods:**

Levels of 8-hydroxy-deoxyguanosine (8-OHdG), 3-nitrotyrosine (3-NT), glutathione (GSH), superoxide dismutase (SOD) and Interleukin-6 (IL-6) were estimated before and after 2 weeks of insulin therapy in normal group (NC) and type 2 diabetic (DM) with normal albuminuria (NA), microalbuminuria (MA) and clinical albuminuria (CA).

**Results:**

In DM group, levels of 8-OHdG and 3-NT were higher than those in NC group (*P* < 0.05); GSH and SOD were lower (*P* < 0.05). And their levels changed with urine albumin–creatinine ratio (*P* < 0.05). After insulin therapy, these derangements were significantly ameliorated and the changes in NA and MA groups were more significant than CA group (*P* < 0.05). Correlation analysis showed glycated hemoglobin, the course of disease, the HOME-IR and fasting plasma glucose were positively correlated with 8-OHdG and 3-NT, but negatively correlated with GSH and SOD.

**Conclusions:**

The oxidative stress gradually increased with the magnitude of DN, and insulin pump intensive therapy can significantly ameliorate the derangements in the early stage of DN.

*Trial registration* NCT03174821

## Background

Type 2 diabetes mellitus (T2DM) is a multifactorial and genetically heterogeneous disease which is characterized by insulin hypo-secretion and/or insulin resistance (IR). The total number of people with diabetes is projected to rise from 171 million in 2000 to 366 million in 2030 [1]. About 30% of type 1 diabetes mellitus (T1DM) and 20–50% of T2DM patients suffered DN, which account for one-third of all dialysis and renal allograft sufferers [2]. Nephropathy is the major cause of disability and death among people suffering diabetes mellitus. However, the molecular mechanisms responsible for its development are, as yet, incompletely understood.

The recent study [3] suggested that oxidative stress resulting from increased production of ROS plays a crucial role in the development of diabetic complications. Oxidative stress is recently recognized as a major factor of cellular damages caused by hyperglycemia [4]. Some studies [5, 6] also have demonstrated that oxidative stress is correlated to the average blood glucose concentration within 24 h and glycosylated hemoglobin (HbAlc). These carbonyl compounds form covalent adducts with specific lysine and arginine residues in proteins and intracellular advanced glycation end products (AGEs), which consequently alter protein function. Several large randomized controlled trials have conclusively demonstrated that early strict glycemic control can significantly decrease the risk of DN but increase the risk of the accidence of hypoglycemia [7, 8]. Insulin pump therapy is better than subcutaneous injection in terms of the drug safety and effectiveness. Furthermore, it can effectively avoid the insulin resistance and increase the sensitivity by human insulin preparations [9].

Above all, in order to understand the effect of oxidative stress on diabetic nephropathy and to further precede the clinical valid treatment measures, we monitored the level of oxidative stress of patient in different stages of diabetic nephropathy before and after insulin pump therapy.

## Patients and methods

### Subjects

A total of 80 type 2 diabetes patients and 80 non-diabetic age- and gender-matched subjects were randomly collected between October 2010 and June 2012 from the military general hospital of Beijing. Diagnosis of diabetes was made according to the criteria of the World Health Organization by Endocrinology Department of the military general hospital of Beijing (patients were excluded if they had received a diagnosis of type 1 diabetes). Male to female ratio was 7:9. Mean age was 54.1 ± 7.4 years (range 40–70). The study was carried out in compliance with the Institutional Ethics Committee and informed consent being given to each participant. The inclusion criterion and exclusion criterion are described in Table 1.

**Table 1:** The inclusion and exclusion criteria of this study subjects

Inclusion criterion	Fasting plasma glucose (FPG) ≥ 10 mmol/l and/or 2-h plasma glucose (2hPG) ≥ 15 mmol/l
Exclusion criterion	(1) Using antioxidant drugs within 1 month
	(2) Accompanied with acute and chronic severe complications, fever, malignant tumor, nephritis, and congestive heart failure
	(3) Accompanied with diabetic ketosis, ketoacidosis, severe hypoglycemia, and hyperosmolar hyperglycemic state
	(4) With other endocrine diseases, autoimmune diseases or connective tissue diseases
	(5) Having history of infection within 1 month
	(6) Having drug or alcohol dependence
	(7) Severe hypoxia and stress state (e.g., cardiovascular events, trauma, surgery, and consumptive disease)

DN status was determined by measurements of urinary albumin-to-creatinine ratio (UACR). The 80 T2DM patients were divided into three sub-groups: normal albuminuria (NA), microalbuminuria (MA) and clinical albuminuria (CA) and then were given 2-week insulin pump therapy. 80 healthy persons were randomly chosen as normal group (NC), male to female ratio was 31:49 and mean age 53.1 ± 7.0 years (Table 2).

**Table 2:** The group were divided by UACR

Groups	UACR (mg/g)	Total
Normal albuminuria (NA) (*n* = 35)	UACR < 30	*n* = 80
Microalbuminuria (MA) (*n* = 30)	30 ≤ UACR < 300	
Clinical albuminuria (CA) (*n* = 15)	UACR ≥ 300	
Normal group (NC) (*n* = 80)	Non-diabetic subjects	*n* = 80

### Pre-therapy detection

All patients underwent the physical examination, which included height (cm), weight (kg), blood pressure (mmHg) and body mass index (BMI, kg/m^2^). Venous blood samples were drawn from all subjects in the fasting at least 8 h. (1) The fasting plasma glucose were measured by means of glucose oxidase method (Glucose GOD FS, DiaSys Diagnostic Systems GmbH, Germany). (2) Determination of hepatic and renal function, total cholesterol (TC), triglyceride (TG), high-density lipoprotein cholesterol (HDL-C), low-density lipoprotein cholesterol (LDL-C) and HbAlc was performed with automatic biochemistry analyzer (HITACHI 7020, Japanese), at the same time determining the blood C-reactive protein (CRP). (3) Fasting serum insulin (FINS) was determined by immunoluminometric assay detected by BECKMANCOULTER-ACCESS, American, and then the homeostasis model assessment-insulin resistance (HOMA-IR) was calculated by the formula: HOMA-IR = FINS × FPG/22.5. 5. The hepatic duct extracted 5 ml venous blood, precipitated, centrifuged and took the serum in the − 70 °C refrigerator to detect the level of the 3-NT, 8-OHdG, SOD, GSH (Elisa kit, Shanghai Jiang Lai Biological Technology Co. China) and IL-6 (PD6050, R&D Systems) by enzyme-linked immune competition assay.

First morning mid-stream clean urine (5 ml) was sent to endocrinology laboratory and the determination of urine albumin/creatinine (A/C) was measured by immune turbidimetry. 2-h plasma glucose (2hPG) was detected by standardized steamed bread meal test.

### Insulin pump therapy

Initial dose calculation: the total requisite amount = 0.44 × weight (kg). The preprandial and basal amount, respectively, took up to 50% of integral dose. 15 min before meal, the preprandial insulin was equally given by three times. The basal insulin was pumped at 00:00–3:00, 3:00–8:00, 8:00–14:00, 14:00–20:00 and 20:00–24:00. The dosage of insulin given at 00:00–3:00 was the least to avoid the hypoglycemia, and the dosage at 3:00–8:00 was the maximum to deal with diabetes mellitus dawn phenomenon (about 150–200% of dosage at period of 00:00–3:00). Others were between these. The rapid blood glucose was measured at 3:00, 6:00, 9:30, 11:00, 14:00, 16:30, 19:00 and 21:00. If there was glycopenia in the patient, we would measure and record another blood sugar. The bolus and basal rates work to keep blood sugars constantly in control (FPG: 4–6 mmol/l, 2hPG: 5–8 mmol/l). After 2 weeks, all patients finished insulin pump therapy; the blood sugar, insulin and other biochemical indexes were tested next morning. The rest blood serum was kept in the − 70 °C refrigerator and the level of 3-NT, 8-OHdG, SOD, GSH and IL-6 was measured by enzyme-linked immune-sorbent assay. In the whole treatment period, all the subjects insist on the dietary therapy and physical exercise treatment, but without any oral hypoglycemic agents.

### Statistical analysis

Statistical analyses were performed using software of SPSS 17.0. The measurement data were expressed as the mean ± SD. The results of before and after treatment with insulin pump were applied comparative *t* test to perform statistical analysis. The inter-group comparison was performed by one-way analysis of variance (ANOVA) methodology. The enumeration data were statistically analyzed by Chi square test. The correlation analysis between two factors was undertaken with simple linear correlation analysis. *P* < 0.05 was considered significant.

## Results

### Baseline parameters

Baseline parameters of all the participants in this study are presented in Table 3. There were no significant differences at baseline in terms of age, gender and diastolic blood pressure (DBP). The level of BMI, FPG, 2hPG, HbA1c, TG, TC, LDL-C, 8-OHdG, 3-NT, CRP, SBP and IL-6 in DM group was higher than that in NC group, and the level of GSH, SOD and HDL-C was lower than NC group, both of the differences were significant (*P* < 0.05).

**Table 3:** Baseline characteristics of DM and NC groups

	Groups		*T* value*/*χ^2^	*P*
	NC	DM		
Gender (M/F)	31/49	35/45	0.413	0.630^☆^
Age (years)	53.1 ± 7.0	54.1 ± 7.4	− 0.845	0.4
BMI (kg/m^2^)	23.8 ± 1.3	24.7 ± 2.0	− 3.465	0.001*
SBP (mmHg)	127.1 ± 9.1	132.3 ± 12.1	− 3.097	0.002*
DBP (mmHg)	77.2 ± 6.2	77.4 ± 5.4	− 0.284	0.777
HBA1c (%)	5.2 ± 0.4	10.6 ± 1.5	− 30.098	0.000*
FPG (mmol/l)	4.6 ± 0.6	12.4 ± 1.8	− 38.143	0.000*
2hPG (mmol/l)	5.7 ± 0.4	16.5 ± 1.2	− 76.735	0.000*
HOMA-IR	1.4 ± 0.3	3.3 ± 0.5	− 26.932	0.000*
IL-6 (pg/l)	94.7 ± 11.2	167.90 ± 15.46	− 34.292	0.000*
TC (mmol/l)	4.0 ± 0.7	5.0 ± 1.0	− 7.198	0.0008*
TG (mmol/l)	1.5 ± 0.2	2.8 ± 0.9	− 13.499	0.000*
LDL (mmol/l)	2.5 ± 0.3	3.3 ± 0.8	− 8.112	0.000*
HDL (mmol/l)	1.4 ± 0.3	1.1 ± 0.4	6.121	0.000*
3-NT (nmol/l)	78.6 ± 7.4	121.5 ± 9.9	− 31.13	0.000*
SOD (U/ml)	124.8 ± 17.6	102.6 ± 20.5	7.332	0.000*
8-OHdG (ng/ml)	6.9 ± 3.8	25.4 ± 4.4	− 28.471	0.000*
GSH (mg/l)	152.5 ± 18.9	134.3 ± 15.3	6.663	0.000*
CRP (mg/l)	2.6 ± 0.7	8.46 ± 1.79	− 27.477	0.000*

^☆^Chi square test, others were *t* test; * *P* < 0.05 compared with the normal control group

### The parameters of DM groups after insulin therapy

It is notable that the patients’ blood pressure, FPG, 2hPG, TG, TC, LDL-C, 8-OHdG, 3-NT, CRP and IL-6 levels were significantly decreased in the DM group after 2-week insulin pump therapy as compared with the levels before treatment (*P* < 0.05). Also, the levels of GSH, SOD and HDL-C were increased after 2-week treatment and the difference was significant (*P* < 0.05). However, HbA1c had no statistically significant differences among the treatments (Table 4).

**Table 4:** The parameters of DM groups at the baseline and at end of insulin pump therapy

	Treatment		*T* value	*P* value
	Pre-	Pro-		
SBP (mmHg)	132.3 ± 12.1	130.5 ± 11.5	− 4.413	*0.336*
DBP (mmHg)	77.4 ± 5.4	75.5 ± 5.8	− 4.905	0.033*
HBA1c (%)	10.6 ± 1.5	10.5 ± 1.5	− 1.85	*0.068*
FPG (mmol/l)	12.4 ± 1.8	7.3 ± 1.0	− 23.006	0.000*
2hPG (mmol/l)	16.5 ± 1.2	9.1 ± 1.1	− 40.791	0.000*
IR	3.3 ± 0.5	2.6 ± 0.4	− 16.508	0.000*
TC (mmol/l)	5.0 ± 1.0	3.4 ± 0.9	− 15.075	0.000*
TG (mmol/l)	2.8 ± 0.9	2.4 ± 0.9	− 4.22	0.000*
CRP (mg/l)	8.46 ± 1.79	6.00 ± 1.60	− 13.14	0.000*
IL-6 (pg/l)	167.90 ± 15.46	106.95 ± 17.80	− 43.08	0.000*
3-NT (nmol/l)	121.5 ± 9.9	114.6 ± 9.5	− 12.064	0.000*
SOD (U/ml)	102.6 ± 20.5	115.0 ± 18.3	19.31	0.000*
8-OHdG (ng/ml)	25.4 ± 4.4	21.3 ± 4.9	− 12.109	0.000*
GSH (mg/l)	134.3 ± 15.3	145.0 ± 14.0	14.946	0.000*
LDL (mmol/l)	3.3 ± 0.8	2.5 ± 0.6	− 7.943	0.000*
HDL (mmol/l)	1.1 ± 0.4	1.3 ± 0.4	4.881	0.000*

* *P* < 0.05 between pre-treatment and pro-treatment in the DM group

### Oxidative stress indicators and inflammatory factor levels among NC group and each DM sub-groups before insulin pump therapy

**Fig. 1:**
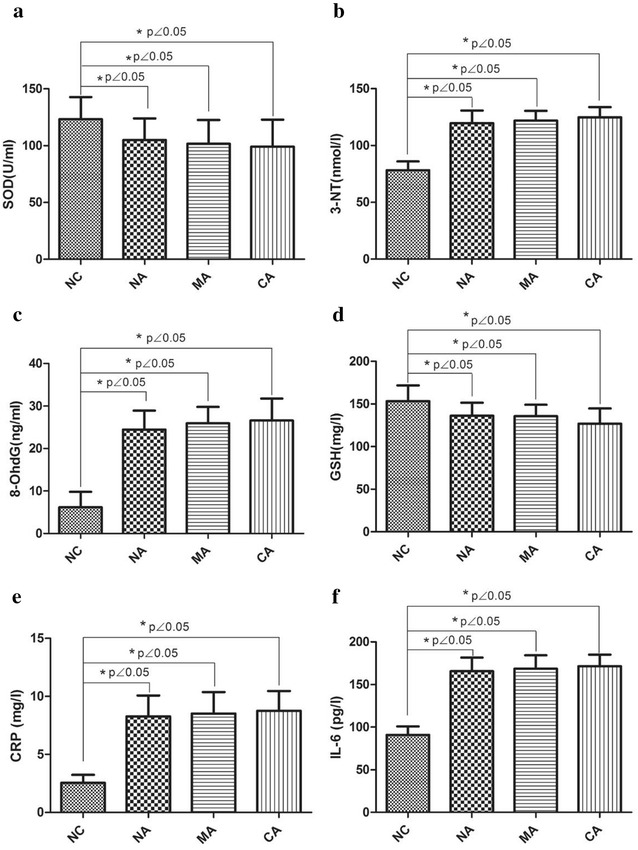
The comparison of SOD (**a**), 3-NT (**b**), 8-OHdG (**c**), GSH (**d**), CRP (**e**) and IL-6 (**f**) levels among NC, NA, MA and CA groups before insulin pump therapy

The levels of 8-OHdG, 3-NT, CRP and IL-6 were reduced in the group of NA, MA and CA compared with NC group (*P* < 0.05), whereas the GSH and SOD levels were significantly raised as shown in Fig. 1. However, the changes among NA, MA and CA groups were not statistically significant (*P* > 0.05, Table 5).

**Table 5:** The parameters of NC group and each DM sub-groups at baseline of insulin pump therapy

Groups	3-NT (nmol/l)	SOD (U/ml)	8-OHdG (ng/ml)	GSH (mg/l)	CRP (mg/l)	IL-6 (pg/l)
NC	78.6 ± 7.4	124.8 ± 17.6	6.9 ± 3.8	152.5 ± 18.9	2.6 ± 0.7	94.7 ± 11.2
NA	119.6 ± 11.2*	104.9 ± 19.0*	24.5 ± 4.4*	136.3 ± 15.2*	8.29 ± 1.80*	165.67 ± 16.11*
MA	122.0 ± 8.4*	101.6 ± 20.9*	26.0 ± 3.9*	135.7 ± 13.4*	8.51 ± 1.85*	168.65 ± 15.69*
CA	124.8 ± 8.9*	99.0 ± 23.7*	26.6 ± 5.2*	126.8 ± 17.9*	8.76 ± 1.70*	171.63 ± 13.40*

* *P* < 0.05 compared with NC group

### Oxidative stress indicators in DM sub-groups before and after insulin pump therapy

**Fig. 2:**
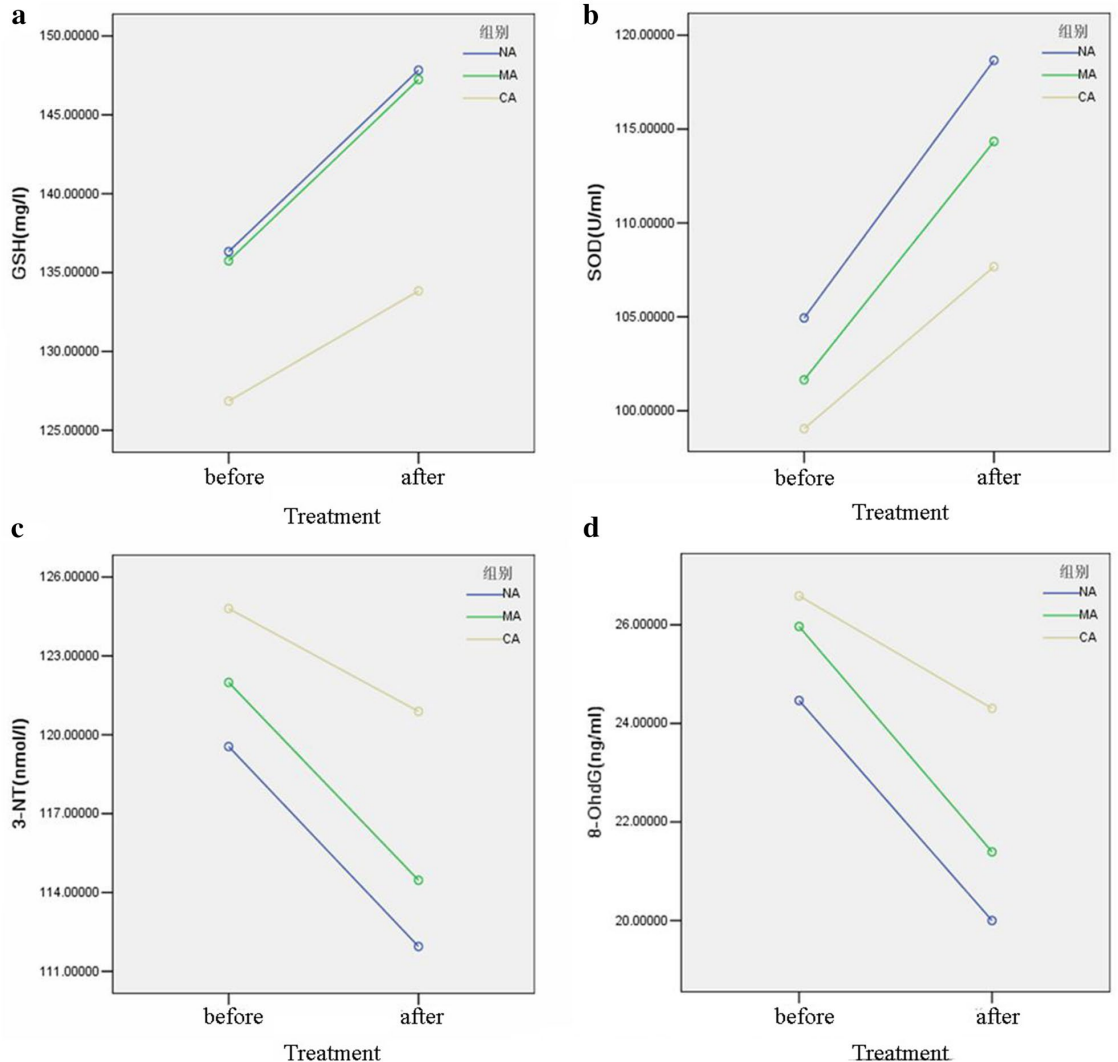
The comparison of the levels of GSH (**a**) and SOD (**b**), 3-NT (**c**) and 8-OHdG (**d**) in NA, MA and CA groups before and after insulin pump therapy

**Fig. 3:**
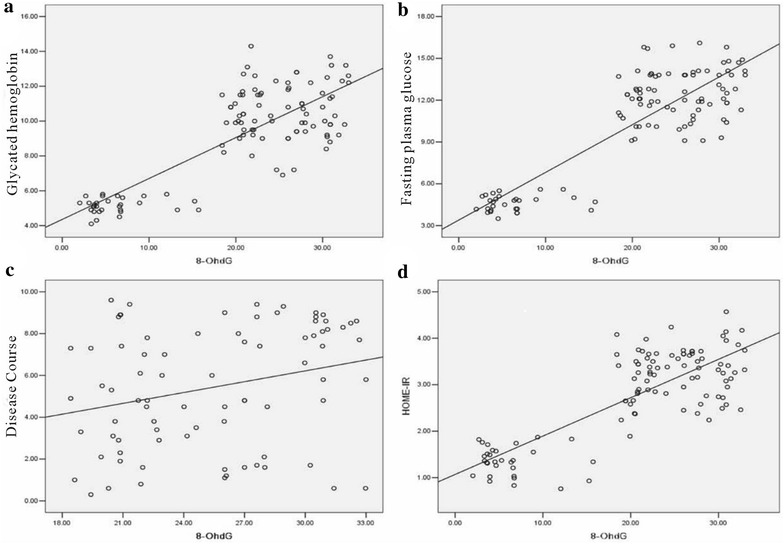
The relation assessment between 8-OHdG and parameters such as glycated hemoglobin (**a**), fasting plasma glucose (**b**), the course of disease (**c**), and the HOME-IR (**d**)

After 2-week insulin pump intensive therapy, the levels of GSH (Fig. 2a) and SOD (Fig. 2b) levels were increased (*P* < 0.05); the levels of 8-OHdG (Fig. 2d) and 3-NT (Fig. 2c) were both lower than that pre-treatment. Furthermore, the differences in the NA and MA group were more significant than that in the CA group (*P* < 0.05) (Table 6).

**Table 6:** The changes of oxidative stress indicators in DM sub-groups at the baseline and at end of insulin pump therapy

Groups	NA	MA	CA	*F* value	*P* value
3-NT (nmol/l)					
Pre-treatment	119.6 ± 11.2	122.0 ± 8.4	124.8 ± 8.9		
Pro-treatment	111.9 ± 9.2	114.5 ± 8.6	120.9 ± 9.8		
*D* value	− 7.6 ± 5.1*	− 7.5 ± 5.0*	− 3.9 ± 4.5	3.316	0.042*
SOD (U/ml)					
Pre-treatment	104.9 ± 19.0	101.7 ± 20.9	99.0 ± 23.7		
Pro-treatment	118.7 ± 17.8	114.3 ± 17.2	107.7 ± 20.4		
*D* value	13.7 ± 4.9*	12.7 ± 6.0*	8.6 ± 5.7	4.588	0.013*
8-OHdG (ng/ml)					
Pre-treatment	24.5 ± 4.4	26.0 ± 3.9	26.6 ± 5.2		
Pro-treatment	20.0 ± 4.0	21.4 ± 4.3	24.3 ± 6.4		
*D* value	− 4.5 ± 2.8*	− 4.6 ± 3.2*	− 2.3 ± 2.7	3.543	0.034*
GSH (mg/l)					
Pre-treatment	136.3 ± 15.2	135.7 ± 13.4	126.8 ± 17.9		
Pro-treatment	147.8 ± 13.1	147.2 ± 9.6	133.8 ± 18.0		
*D* value	11.5 ± 6.2*	11.5 ± 6.7*	7.0 ± 5.2	3.225	0.045*

* *P* < 0.05 comparison with CA group

### The relationship between oxidative stress indicators and other parameters

**Fig. 4:**
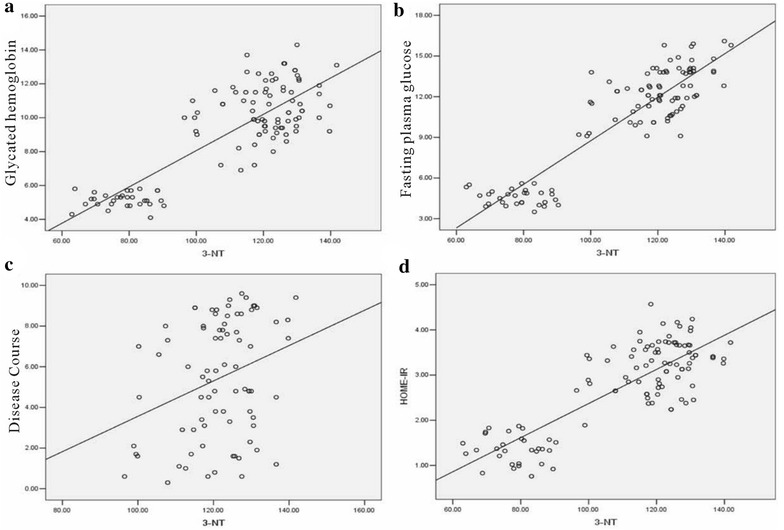
The relation assessment between 3-NT and parameters such as glycated hemoglobin (**a**), fasting plasma glucose (**b**),the course of disease (**c**), and the HOME-IR (**d**)

**Fig. 5:**
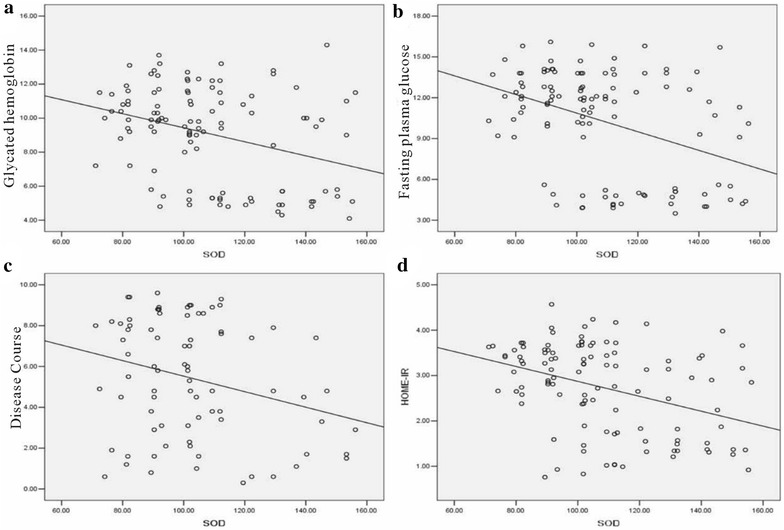
The relation assessment between SOD and parameters such as glycated hemoglobin (**a**), fasting plasma glucose (**b**), the course of disease (**c**) and the HOME-IR (**d**)

**Fig. 6:**
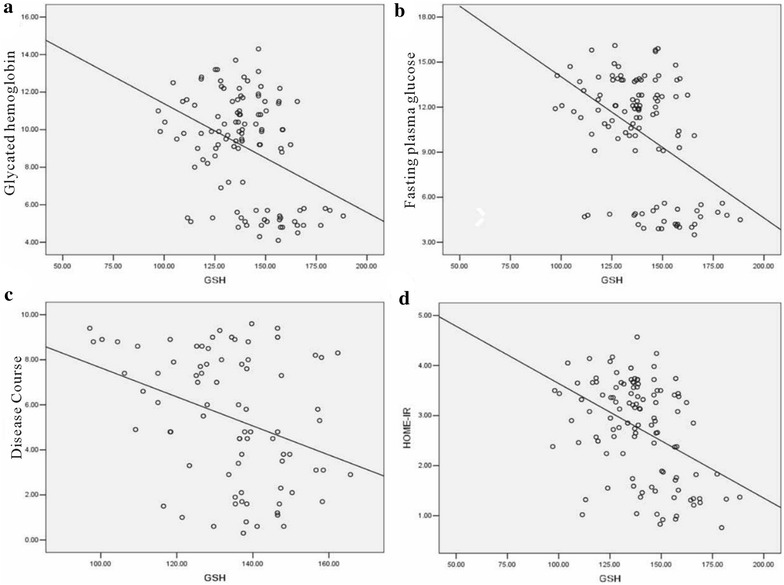
The relation assessment between GSH and parameters such as glycated hemoglobin (**a**), fasting plasma glucose (**b**), the course of disease (**c**), and the HOME-IR (**d**)

To determine the relationship between oxidative stress indicators and other parameters (glycated hemoglobin, the course of disease, the HOME-IR and fasting plasma glucose), we used Pearson relation assessment. We observed that the levels of 8-OHdG and 3-NT were positively correlated with the index of glycated hemoglobin, the course of disease, the HOME-IR and fasting plasma glucose (*P* < 0.05) (Figs. 3, 4; Table 7). As shown in Figs. 5 and 6, a negative association was observed between the levels of GSH and SOD and the index of glycated hemoglobin, the course of disease, the HOME-IR and fasting plasma glucose (the correlation index was − 0.382, − 0.343, − 0.428, − 0.450 for GSH and − 0.330, − 0.271, − 0.372, − 0.396 for SOD in turn, respectively, *P* < 0.05).

**Table 7:** The correlation coefficients of the 8-OHdG, 3-NT, GSH and SOD

	8-OHdG		3-NT		SOD		GSH	
	*r*	*P* value	*r*	*P* value	*r*	*P* value	*r*	*P* value
GH	0.811	0.032	0.829	0.003	− 0.330	0.024	− 0.382	0.035
CD	0.264	0.015	0.298	0.007	− 0.271	0.015	− 0.343	0.026
HOME-IR	0.807	0.022	0.827	0.046	− 0.372	0.004	− 0.428	0.005
FPG	0.857	0.018	0.902	0.002	− 0.396	0.003	− 0.450	0.019

## Discussions

DN is one of the most important microvascular complications of diabetes, including diabetic neuropathy and diabetic retinopathy [3, 10]. Oxidative stress resulting from an imbalance between antioxidants and oxidants lead to cell damage in the harmful irritant [11]. It is well known that the generation of mitochondrial reactive oxygen species (ROS) is the primary initiating event that subsequently activates other pathways implicated in the development of the complications of diabetes [12]. Hyperglycemia has been demonstrated to be the key contributor in the development of DN in both type 1 and type 2 diabetes [13]. On the other hand, persistent hyperglycemia damages cells by four major pathways on the decline of kidney function, namely the hexosamine and polyol pathways, protein kinase C isoforms, and formation of the reactive carbonyl compounds methylglyoxal (MG) and glyoxal [14–16].

The recent study [17] has showed that 8-OHdG was significantly elevated in the diabetic and the pre-diabetic groups (including patients with impaired glucose tolerance (IGT) or elevated fasting glucose) compared with the normal people. And the change of 8-OHdG had an evident heredity [18]. The scientists (Ceriello et al. [27]) proved that with the high concentration of blood sugar, 3-NT was induced by the increased expression of ONOO–, H_2_O_2_, OH and NO, and lead to denatured protein and enzyme, DNA damage and cell apoptosis. GSH combined with glutathione reductive and vitamin C to form a benign circulation of antioxidant process [19]. And by catalyzing the superoxide anion radical to produce disproportionation reaction, SOD maintained the balance of ROS in vivo [20]. Another report [21] illustrated that the condition of high glucose and high fat in the T2DM induced the oxidative stress, and the excess of ROS/RNS also could decrease the level of GSH and SOD. Brownlee stated that as the byproduct of reaction of free radicals with DNA, protein and lipid 8-OHdG, 3-NT can reflect the level of oxygen free radical in the body so as the level of GSH and SOD to the damage of antioxidant system.

Our study found that the level of 8-OHdG and 3-NT in DM group was significantly higher than NC group, and GSH and SOD level is obviously lower than that in NC group. Both of the changes were related to the level of UACR. There is no difference between the antioxidant ability of NA, MA and CA groups, which showed that the enhanced oxidation ability rather than the antioxidant ability of weakening played the leading role in the enhancement of oxidative stress in DN patients. The results in Table 3 suggested that patients of type 2 diabetes mellitus had severe oxidative stress and the ROS became worse in development of DN. Correlation analysis showed that 8-OHdG and 3-NT had positive relation with glycated hemoglobin, the course of disease, the HOME-IR and fasting plasma glucose. The result suggested that abnormal glucose metabolism played an important role in the pathophysiology of DN.

Insulin pump can mimicry the pulsatile secretion of insulin under normal condition in vivo, and we used the INS-pump to properly release insulin in order to maintain the stability of blood sugar in the base and additional quantity. Large-scale clinical trials have confirmed that intensive insulin therapy can provide rapid and effective glycemic control, microcirculation and reduced substances harmful to the kidney, produced by the body in early type 2 diabetes mellitus patients, and thereby reducing the leakage of urine protein, slow the progression of DN [22]. Our study found that after short-term insulin pump therapy, the levels of 8-OHdG and 3-NT were significantly reduced but GSH and SOD were increased in MA, NA and CA groups. The changes were even significant in MA and NA groups than in CA group. These results indicated the critical functions of intensive insulin therapy in suppress oxidative stress.

Numerous studies [23] showed that elevated blood glucose can cause a large amount of ROS, protein glycosylation and glucose autoxidation, leading to the occurence of oxidative stress. Persistent high blood sugar can also activate protein kinase C/NAD(P)H oxidase pathway to increase production of ROS in vivo [24]. There was also research [25] that confirmed hyperglycemia by activation of protein kinase A (protein kinases A, PKA) resulting in decreased production of NADPH and levels of intracellular GSHt, leading to the decreased antioxidant capacity in kidney tissues. In this study, correlation analysis showed 8-OHdG and 3-NT were positively correlated with fasting plasma glucose. The insulin pump therapy can quickly hypoglycemic; we considered use of insulin pump therapy could improve patient oxidative stress through improving insulin resistance and smooth hypoglycemic. In addition, T2DM patients always combine with dyslipidemia characterized by increasing triglyceride (TG) levels and HDL-C decrease [26]. Along with the increasing TG, fatty acids (FFA) were elevated and underwent mitochondrial uncoupling and β oxidation to produce large amount of ROS causing direct damage to the body [27, 28]. The FFA could also destruct pancreatic β cells to reduce insulin secretion, which lead to hyperglycemia and caused body damage [29, 30]. Recent studies [31] showed that inflammation is also involved in the occurrence and development of DN. Persistent chronic hyperglycemia damaged renal cells, the damaged renal cells released inflammatory mediators, and then filtered off and caused leukocytes activation in the site of injury. Renal biopsy specimens in patients with diabetes and diabetic rats were found in the excessive increase of leukocyte adhesion molecules and macrophage infiltration. Leukocytes, granulocytes, monocytes, macrophages and other cells are involved in different inflammatory lesions of DN process [32].

## Conclusions

After the insulin pump therapy, we observed that blood lipids in DM group (except HDL) decreased and inflammatory cytokines’, IL-6 and CRP, levels decreased. Therefore, we conclude that insulin pump therapy may improve the state of the patient’s body by lipid levels and inflammatory state thereby improving the body’s oxidative stress.

Therefore, we advocate to intensive insulin therapy in early DN, in order to obtain greater benefits.

## Abbreviations

DN: diabetic nephropathy
8-OHdG: 8-hydroxy-deoxyguanosine
3-NT: 3-nitrotyrosine
GSH: glutathione
SOD: superoxide dismutase
IL-6: interleukin-6
NC: normal control
DM: diabetes
NA: normal albuminuria
MA: microalbuminuria
CA: clinical albuminuria
SOD: superoxide dismutase
UACR: urine albumin–creatinine ratio
